# Comparison of two protocols for the generation of iPSC-derived human astrocytes

**DOI:** 10.1186/s12575-023-00218-x

**Published:** 2023-09-20

**Authors:** Patrycja Mulica, Carmen Venegas, Zied Landoulsi, Katja Badanjak, Sylvie Delcambre, Maria Tziortziou, Soraya Hezzaz, Jenny Ghelfi, Semra Smajic, Jens Schwamborn, Rejko Krüger, Paul Antony, Patrick May, Enrico Glaab, Anne Grünewald, Sandro L. Pereira

**Affiliations:** 1 Luxembourg Centre for Systems Biomedicine, University of Luxembourg, Esch-Sur-Alzette, Luxembourg; 2 Luxembourg Institute of Health, Strassen, Luxembourg; 3 Institute of Neurogenetics, University of Lübeck, Lübeck, Germany

**Keywords:** iPSC, Astrocytes, Disease modeling

## Abstract

**Background:**

Astrocytes have recently gained attention as key contributors to the pathogenesis of neurodegenerative disorders including Parkinson’s disease. To investigate human astrocytes in vitro, numerous differentiation protocols have been developed. However, the properties of the resulting glia are inconsistent, which complicates the selection of an appropriate method for a given research question. Thus, we compared two approaches for the generation of iPSC-derived astrocytes. We phenotyped glia that were obtained employing a widely used long, serum-free (“LSF”) method against an in-house established short, serum-containing (“SSC”) protocol which allows for the generation of astrocytes and midbrain neurons from the same precursor cells.

**Results:**

We employed high-content confocal imaging and RNA sequencing to characterize the cultures. The astrocytes generated with the LSF or SSC protocols differed considerably in their properties: while the former cells were more labor-intense in their generation (5 vs 2 months), they were also more mature. This notion was strengthened by data resulting from cell type deconvolution analysis that was applied to bulk transcriptomes from the cultures to assess their similarity with human postmortem astrocytes.

**Conclusions:**

Overall, our analyses highlight the need to consider the advantages and disadvantages of a given differentiation protocol, when designing functional or drug discovery studies involving iPSC-derived astrocytes.

**Supplementary Information:**

The online version contains supplementary material available at 10.1186/s12575-023-00218-x.

## Introduction

Astrocytes constitute the largest cell population among glial cells residing in the mammalian brain and play a crucial role in maintaining its proper functioning [1]. Astrocytes are involved in synapse formation, the regulation of brain blood flow, ion and neurotransmitter homeostasis and, most importantly, metabolic support of neuronal functions [2]. Moreover, astrocytes are acknowledged as being critical for the regulation of neuroinflammation, with their ability to recognize inflammatory signals and subsequently react to them through the production of numerous chemokines and cytokines [3–5].

Astrocytes originate from neural stem cells, also known as radial glia [6]. Astrocytic development is a highly regulated process, which is initiated after neurogenesis and controlled by a myriad of intrinsic and extrinsic factors [7]. Key cues for the initiation of gliogenesis are the JAK-STAT and Notch signaling pathways [8, 9]. The initiation of the JAK-STAT pathway is crucial at the onset of astrogenesis and is controlled by changes in the sequestration of p300/CBP, its key activator [10]. Moreover, extrinsic factors such as FGF2 and retinoic acid can activate the pathway by triggering chromatin remodeling [11]. Importantly, cytokines of neuronal origin such as CT-1, LIF, CNTF, and BMPs can further influence the activity of the JAK-STAT pathway [6]. Astrogenesis is also orchestrated by the Notch pathway, which is activated by emerging neurons [12]. Both JAK-STAT and Notch signaling regulate each other thereby contributing to the complexity of astrocyte generation [6]. After having populated different regions of the central nervous system, astrocytic precursors begin to mature, i.e. they acquire an expression profile typical for fully differentiated astrocytes. In addition to the upregulation of the established astrocyte marker *GFAP*, the mature state of astrocytes was correlated with the overexpression of genes such as *S100B*, *SLC1A2*, and *ALDH1L1*. Furthermore, astrocytes develop highly branched processes, which form non-overlapping domains and their proliferation ceases [8, 13, 14].

Recently, astrocytes have gained attention as key contributors to the pathogenesis of numerous neurodegenerative diseases [5]. One of the hallmarks of these disorders is an astrocytic transition from a resting to a reactive state [15, 16]. Reactive astrogliosis is a term coined to describe transcriptional, biochemical, physiological, and morphological changes that astrocytes undergo when facing brain pathology. Specifically, they may increase *GFAP* levels and drastically modify the morphology of both their soma and processes [17]. Furthermore, reactive astrocytes change their transcriptome [4], in particular their cytokine expression and secretion profiles [3]. Importantly, changes in activation status might exert a profound effect on astrocytic physiological functions, including their crucial role in energetic coupling with neurons [18].

In light of these transformations during glial activation, reliable models that are non-reactive under control conditions must be implemented when unraveling the impact of disease-associated mutations or exposures on astrocyte function in the context of neurodegeneration. This is especially true as drug discovery approaches increasingly rely on iPSC-derived cells given the limitations of rodent models in mirroring key phenotypes of neurodegenerative disorders such as Alzheimer’s (AD) and Parkinson’s disease (PD). Since the advent of iPSC technology, numerous protocols have been developed for the generation of patient-derived astrocytes [19, 20]. Thereby two major types of differentiation from iPSCs are being distinguished: embryoid body and monolayer approaches. In addition to key growth factors, some protocols require the use of fetal bovine serum (FBS), while others are based on the application of small molecules to initiate astroglial differentiation [20]. Moreover, protocols for the direct conversion of fibroblasts into astrocytes have also been established [21]. Previous comparisons revealed that the available protocols vary substantially in terms of labor intensiveness, yield and maturation status of the resulting cells [22], making it difficult to opt for the appropriate protocol for a given research question [20, 23].

Here, we aimed to explore two astrocyte differentiation paradigms, which are substantially different in terms of media composition and duration of the procedure. We compared one of the most widely used differentiation protocols—a long, serum-free (LSF) approach established by Oksanen et al. [24] that represents a slightly modified version of Krencik and colleagues’ method paper [25]—against a short, serum-containing (SSC) differentiation protocol that was previously established in-house [26]. The protocol by Palm and colleagues is of particular interest in PD research as it is based on the use of homogeneous neural stem cells which present robust expandability allowing for the reliable generation of astrocytes and midbrain dopaminergic neurons starting from the same precursor cells [26–30]. By applying RNA sequencing (RNA-seq) and high-content imaging, we were able to thoroughly assess maturity and activation status of the obtained astrocytes. Additionally, we compared iPSC-derived astrocytes with their postmortem human counterparts to gain a better understanding of the suitability of the generated cultures for disease modeling.

## Materials and methods

### Cell culture

Human iPSC lines CTRL1 and CTRL2, which were previously generated as described [31, 32], were maintained in mTeSR™ Plus medium. Astrocytes differentiated directly from iPSCs (herein referred to as “LSF method”) were generated as previously described [24, 25]. In short, iPSCs were converted to neuroepithelial cells by maintenance for 11 days in neurodifferentiation medium (NDM) containing 10 µM SB431542 (Sigma) and 200 nM LDN-193189 (Sigma). NDM consisted of DMEM-F12 (Gibco) and Neurobasal (Gibco) in 50:50 ratio supplemented with 1% B27 without vitamin A (Gibco), 0.5% N2 (Gibco), 1% GlutaMAX (Gibco) and 0.5% penicillin/streptomycin (Gibco). Next, cells were kept for two additional days in NDM supplemented with 25 ng/ml bFGF (Peprotech). Subsequently, cells were dissociated by scraping, plated on ultra-low attachment plates (Corning) and grown for two days in NDM without the addition of growth factors. Under these conditions, cells formed spheres and were maintained in astrodifferentation medium (ADM) for five months, and manually dissociated by cutting once per week. ADM was comprised of DMEM-F12 supplemented with 1% non-essential amino acids (Gibco), 1% N2, 1% GlutaMAX, 0.5% penicillin/streptomycin, 2 µg/ml heparin (Sigma), as well as 10 ng/ml bFGF and 10 ng/ml EGF (Peptrotech). Terminal differentiation was achieved by dissociating the spheres with accutase (Merck Millipore) and plating them on matrigel (Corning)-coated plates, followed by cultivation for 7 days in ADM containing 10 ng/ml CNTF (Peprotech) and 10 ng/ml BMP4 (Peprotech). To obtain biological replicates, spheres were kept separately for at least 1 month and terminal differentiations were started independently.

The alternative two-step protocol referred to as “SSC method” is initiated by the conversion of iPSCs into neural precursor cells (NPCs), by means of dual-SMAD inhibition and induction of WNT and SHH signaling. These cells correspond to neuroepithelial cells that retain the potential to give rise to neural tube and neural crest lineages [32]. After conversion, NPCs were expanded in N2B27 medium consisting of DMEMF-12 (Gibco)/Neurobasal (Gibco) in 50:50 ratio, supplemented with 1% B27 without vitamin A (Gibco), 0.5% N2 (Gibco), 1% GlutaMAX (Gibco) and 1% penicillin/streptomycin (Gibco). Additionally, 3 µM CHIR99201 (Sigma), 0.75 µM purmorphamine (Sigma) and 150 µM ascorbic acid (Sigma) were added to the medium. The second step in this protocol corresponds to the generation of astrocytes from NPCs, as published before [26]. In brief, NPCs were plated and kept in the standard medium for two days. Afterwards, the medium was changed to N2B27 medium containing 3 µM CHIR, 0.75 µM Purmorphamine (PMA), 150 µM ascorbic acid and 20 ng/ml bFGF (Peprotech) for two additional days. At day four, cells were dissociated using accutase and plated in DMEMF-12 supplemented with 1% penicillin/streptomycin, 1% GlutaMAX, 1% N2, 2% B27 with vitamin A (Gibco), 40 ng/ml EGF (Peprotech), 40 ng/ml bFGF and 1.5 ng/ml hLIF (Peprotech). Cells were maintained in this medium for three passages and for terminal differentiation grown in DMEM-F12 containing 1% penicillin/streptomycin, 1% GlutaMAX and 1% FBS (Gibco) for 60–67 days. Biological replicates were prepared by growing and splitting precursor cells separately and subsequently, for each replicate an independent terminal differentiation was conducted.

### Immunocytochemistry and image analysis

To perform immunocytochemistry analysis, cells were fixed using 4% paraformaldehyde in PBS (ThermoScientific). Subsequently, cells were permeabilized and blocked with 0.25% Triton X-100 and 1% BSA in PBS for 1 h. The same solution was used to prepare dilutions of primary and secondary antibodies. Primary antibodies were incubated overnight at four degree at the given dilutions: Oct4 (Abcam, ab19857, 1:1000), TRA-1–60 (Merck Millipore, MAB4360, 1:1000), Sox2 (Santa Cruz Biotechnology, sc-365823, 1:1000), Nestin (Novus, MAB1259, 1:1000), Musashi1 (Abcam, ab21628, 1:500), Pax6 (Imtec Diagnostics, 901,301, 1:1000), Sox1 (R&D Systems, AF3369, 1:100), GFAP (Dako, z0334, 1:500), Vimentin (Abcam, ab24525, 1:500). On the following day, after several washing steps, secondary antibodies were applied for three hours and subsequently washed again with PBS. Afterwards, nuclei were stained with 20 µM Hoechst (LifeTechnologies), cells were washed and subjected to imaging. Images of iPSCs and NPCs were acquired with a Zeiss Axio Imager M2, whereas astrocytes were imaged using a Yokogawa CV8000 microscope.

To perform image analysis of astrocytes in a quantitative manner, custom-made code was prepared using Matlab 2020a. The analysis was run using the High-Performance Computing Platform available at the University of Luxembourg. The code can be shared upon request of the computer vision scripts with IDs 906, 907 and 2352–2354 (contact person: Dr. Paul Antony). Briefly, cellular morphometrics were quantified based on nuclei and GFAP signals. GFAP reporter fluorescence intensity signals were quantified in the perinuclear zone of single cells. Morphometric features from the GFAP channel were analyzed by segmenting soma and GFAP^+^ protrusions and extracting multiple shape descriptors including perimeter, area, and their ratio. Mean intensity for Vimentin signal was quantified inside of the segmented cellular mask.

### RNA-seq analysis

RNA extraction was carried out with the RNeasy Plus Kit (Qiagen) following the manufacturer’s instructions. Library preparation and sequencing were performed by the Beijing Genomics Institute (BGI) in Copenhagen, Denmark, using the BGISEQ-500 platform.

Raw RNA-seq reads were quality-filtered with the removal of adaptor sequences and contaminating low-quality reads. Furthermore, the base percentage distribution and distribution of quality scores along the reads was checked. Data was subsequently pre-processed, applying the software package "Rsubread" [33]. Gene level differential expression analysis to compare the two astrocytic protocols, LSF and SSC, was conducted in the R statistical programming software using the package "DESeq2" [34]. Genes with low expression counts were excluded with the “filterByExpr-function” using the package edgeR [35] with default parameters. To determine *P*-value significance scores for differential expression we used the Wald test followed by an adjustment for multiple hypothesis testing with the Benjamini and Hochberg method [36]. Heat maps were generated using the “heatmap.2” function from the R package "gplots" [37].

Pathway enrichment analyses were performed in the GeneGo MetaCore™ software [38] using a standard enrichment analysis workflow. As input, the gene level differential expression analysis results were used. The statistics for pathway over-representation analysis, including false-discovery rate (FDR) scores based on the method by Benjamini and Hochberg, were calculated for the GeneGo collections of cellular pathway maps, process networks, and Gene Ontology gene set terms.

### Deconvolution analysis

Deconvolution analysis of bulk RNA-seq data was conducted using the “Multi-subject Single Cell deconvolution” method (MuSiC [39]). MuSiC uses cross-subject cell type-specific gene expression from single-cell RNA sequencing (scRNA-seq) data to estimate the relative cell type composition in bulk RNA-seq data. We used three different reference scRNA-seq datasets that were downloaded from the Gene expression Omnibus (GEO): (i) single-nuclei RNA seq (snRNA-seq) from *substantia nigra* and cortex of five control human donors [40] (GSE140231), (ii) snRNA-seq of postmortem midbrain of six controls and five idiopathic PD cases [41] (GSE157783) and (iii) sc-RNAseq data of human embryo ventral midbrain cells between 6 and 11 weeks of gestation [42] (GSE76381). Cell type annotations included in the published metadata were used as reference for cell type proportion inference.

### Quantitative PCR

After RNA isolation, cDNA was synthesized using SuperScript III Reverse Transcriptase Kit (Invitrogen). To perform quantitative PCR (qPCR), PowerTrack SYBR Green Master Mix (Thermofisher) was used and the reaction was run on the LightCycler 480 (Roche), with the primer annealing step at 60 degree. The expression of the genes of interest was normalized to the housekeeping genes *ACTB* and *L27*.

### Statistical analysis

To perform statistical analyses, GraphPad Prism (version 9.4.0) was used. Typically, two-way ANOVA was applied for grouped analysis. Differences with a p-value below 0.05 were considered statistically significant.

## Results

### Astrocytes generated using the LSF protocol resemble morphologically mature astrocytes

**Fig. 1:**
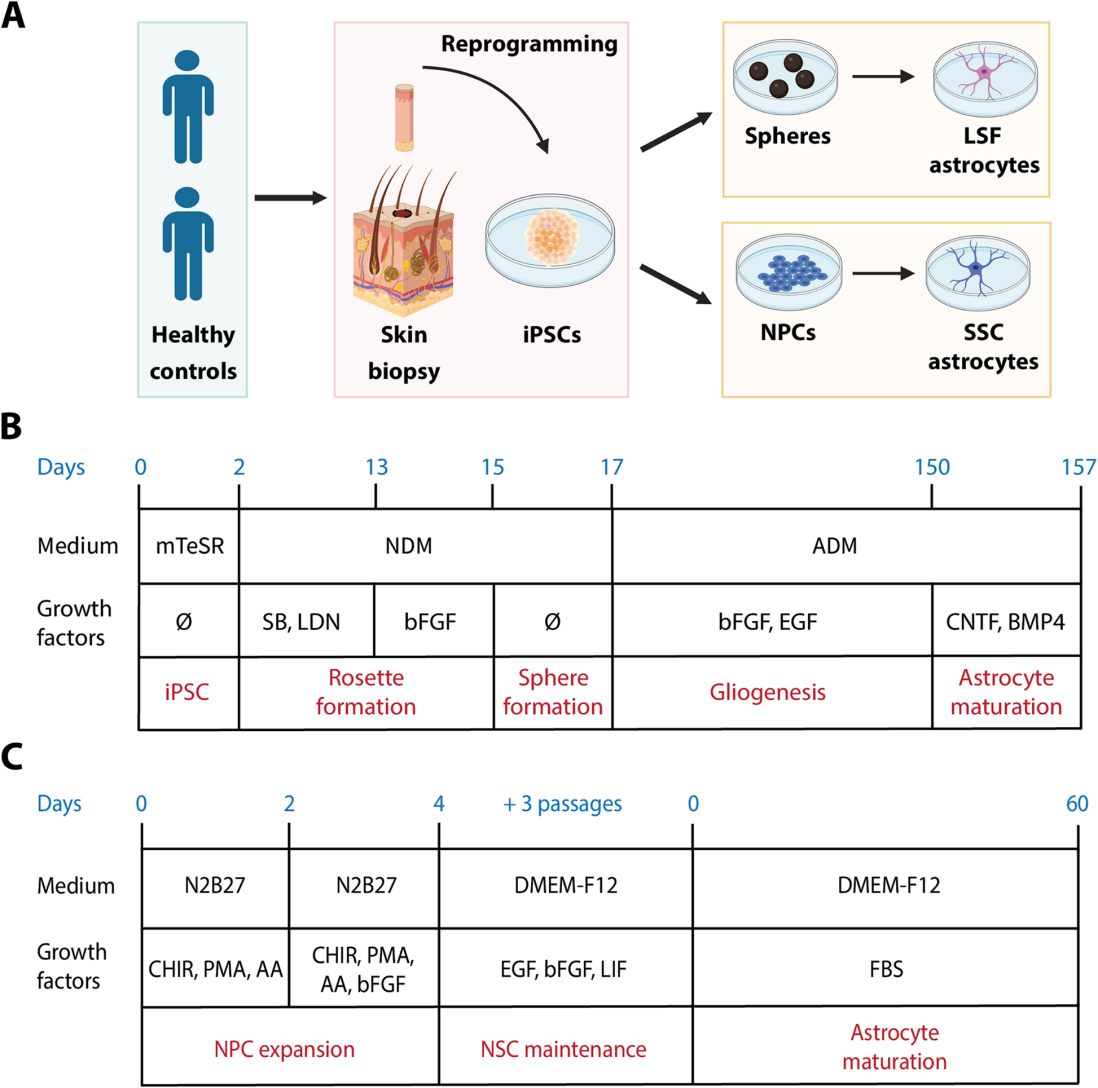
Generation of the astrocytic cultures in this study. **A**. Scheme representing the main steps required to obtain astrocytes with the different protocols. **B**. Overview of the LSF protocol. NDM, neurodifferentiation medium (see materials and methods section for details); ADM, astrodifferentiation medium (see materials and methods section); SB, SB431542; LDN, LDN-193189; CNTF, ciliary neurotrophic factor; BMP4, bone morphogenetic factor 4. **C**. Overview of the SSC protocol. N2B27, medium comprised of DMEM-F12, neurobasal medium and supplements (see material and methods section), CHIR, CHIR99021; PMA, purmorphamine; AA, ascorbic acid; EGF, Epidermal Growth Factor; bFGF, basic fibroblast growth factor; LIF, leukemia inhibitory factor; FBS, fetal bovine serum; NPCs, neural precursor cells; NSCs, neural stem cells. The figure was generated using Biorender

Despite having been neglected for decades, the key role of astrocytes in neurodegeneration has become increasingly evident [43]. To study astrocytic involvement in pathological mechanisms in more detail, disease modeling using iPSC-derived cultures has become a mainstream procedure. However, with the constantly growing number of available protocols [20, 23], the question remains how to select the right approach for a particular research question. To address this point, we applied two distinct protocols to generate iPSC-derived astrocytes (Fig. 1A) from two healthy control lines. The first protocol, here referred to as “long, serum-free” (LSF) protocol”, is based on the generation of neuroepithelial cells, which grow as spheres, and after prolonged maintenance in the presence of EGF and bFGF and manual weekly titration give rise to glial progenitors. As a final step, cells are terminally differentiated by inducing the JAK-STAT pathway and BMP signaling with CNTF and BMP4, respectively [24, 25] (Fig. 1B). The second method, denominated here as “short, serum-containing” (SSC) protocol, utilizes midbrain-specific neural stem cells, which present neuroepithelial features and retain the ability to generate neural tube and neural crest lineages. Accordingly, astrocytes as well as midbrain dopaminergic neurons can be robustly generated starting from the same cultures. Astrocytic differentiation is achieved by cultivation in a medium containing fetal bovine serum (FBS) [26] (Fig. 1C).

Firstly, we characterized all iPSC and neural precursor cell (NPC) lines used in the study to ensure their differentiation potential. The iPSC lines did not show any chromosomal aberrations (Figure S1) and thus were employed for the generation of astrocytes. Furthermore, we assessed the expression of several iPSC and NPC markers using immunocytochemistry. Both iPSC lines expressed typical pluripotency markers such as Oct4, TRA-1–60 and Sox2 (Figure S2A). Moreover, the expression of neural progenitor cell markers, such as Musashi1, Nestin, Pax6 and Sox1, was identified in the NPC lines (Figure S2B) rendering them suitable for differentiation.

**Fig. 2:**
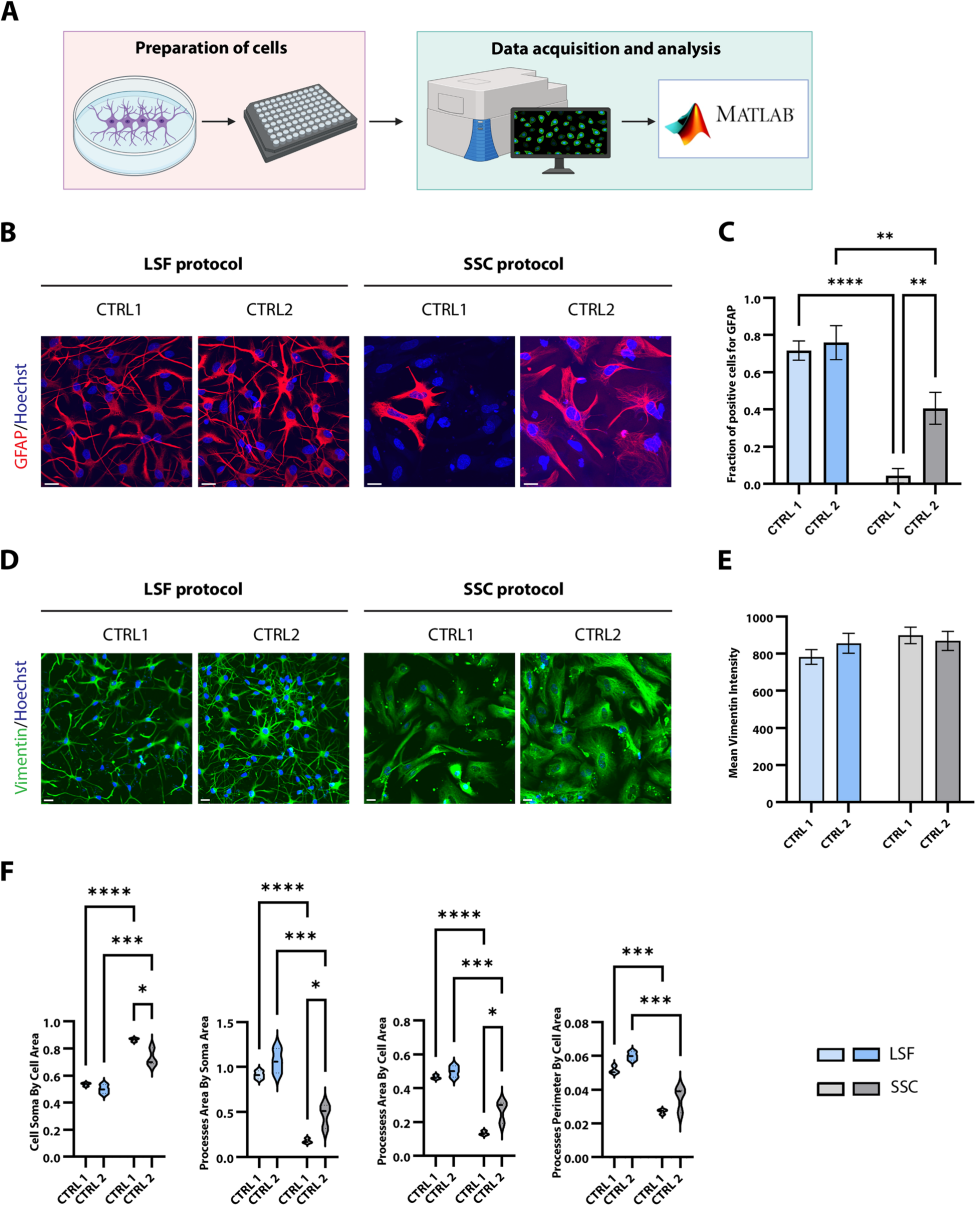
Morphological analysis of the generated astrocytes. **A**. Scheme summarizing the experimental procedure. **B**. Representative images for the late astrocytic marker GFAP for both protocols. The brightness and contrast for the image of CTRL2 in the SSC protocol was adjusted. **C**. Quantification of GFAP^+^ cells in astrocytic cultures using high-content imaging. For each marker, protocol and cell line, three biological replicates were used. Data are presented as the mean ± SD. **D**. Representative images for the early astrocytic marker Vimentin for both protocols. Scale bar: 20 µm. **E**. Quantification of the mean intensity of the Vimentin signal within the segmented cellular mask. Data are presented as the mean ± SD. **F**. Assessment of astrocytic morphology in GFAP^+^ cells. The summed area of astrocytic soma was normalized to the summed cell area. The summed area of astrocytic processes was normalized to the summed soma area. The summed area of astrocytic processes was normalized to the summed cell area. The summed perimeter of astrocytic processes was normalized to the summed cell area. *P ≤ 0.05, **P ≤ 0.01, ***P ≤ 0.001, ***P ≤ 0.0001. Scale bar: 20 µm. CTRL1, control 1; CTRL2, control 2; SD, standard deviation. Part of the figure was generated using Biorender

Next, astrocytes were generated applying the LSF and SSC protocols to iPSCs and NPCs, respectively. The obtained cultures were characterized by employing high-content imaging and custom-made scripts prepared in Matlab (Fig. 2A). This analysis revealed reduced levels of the astrocyte marker GFAP in SSC cultures of both control lines (Fig. 2B, C). To account for the fact that GFAP expression correlates with astrocyte maturation, we additionally performed immunocytochemistry and imaging with an antibody against Vimentin, which is more abundant in immature astrocytes [44]. Contrary to GFAP, this approach indicated comparable intensities between all investigated conditions and lines (Fig. 2D, E). In addition, we assessed the morphology of more mature GFAP^+^ astrocytes. This analysis revealed that LSF astrocytes differ substantially in their morphology from SSC astrocytes. After preparing a Matlab code, which specifically recognized soma and astrocytic processes, we quantified multiple morphological features. Interestingly, LSF astrocytes consistently presented longer and finer branches and a smaller somal area (Fig. 2F). Overall, cells generated with this protocol more closely resembled the prototypical astrocyte morphology [45].

### LSF astrocytes present more mature expression profile

**Fig. 3:**
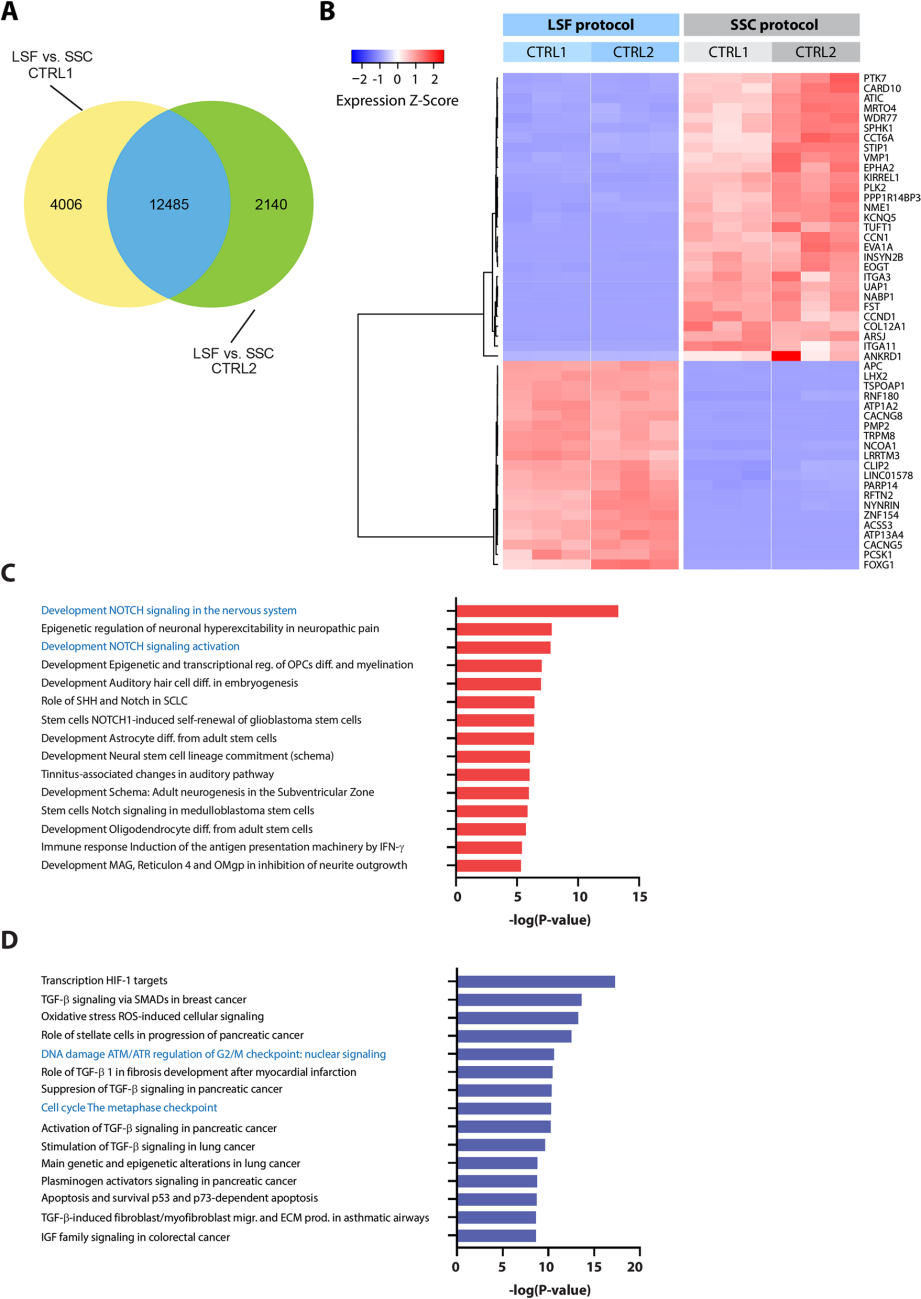
Transcriptomic analysis of astrocytes. **A**. Venn diagram showing the number of overlapping differentially expressed genes (DEGs) when comparing both differentiation protocols. **B**. Heatmap showing 50 top differentially expressed genes between astrocytes obtained with the LSF vs SSC protocols. **C**. Pathway enrichment analysis showing the upregulated pathways in the LSF protocol. **D**. Pathway enrichment analysis showing the downregulated pathways in the LSF protocol

To thoroughly evaluate the differences between astrocytes generated using the LSF and SSC protocols, we utilized bulk RNA-seq. First, we compared differentially expressed genes between the two protocols for each line separately. The analysis revealed in total 16,491 differentially expressed genes, when comparing CTRL1 in the two methods and 14,625 genes for the comparison of CTRL2. Altogether, we identified 12,485 genes, which were differentially expressed between the two protocols for both cell lines (Fig. 3A). Moreover, we plotted the expression Z-score values of the top 50 differentially expressed genes as a heatmap. Among the upregulated genes in the SSC astrocytes, we identified several genes involved in the regulation and promotion of cell proliferation such as *PLK2* [46] and *CCN1* [47] (Fig. 3B), which might suggest a lower degree of maturation of these cells.

To gain more mechanistic insight into the obtained datasets, we performed pathway enrichment analysis. Among the upregulated pathways in LSF astrocytes, we detected Notch signaling (Fig. 3C), which is known to be involved in gliogenesis [48]. Contrariwise, these cells down-regulated pathways linked to cell cycle regulation (Fig. 3D).

**Fig. 4:**
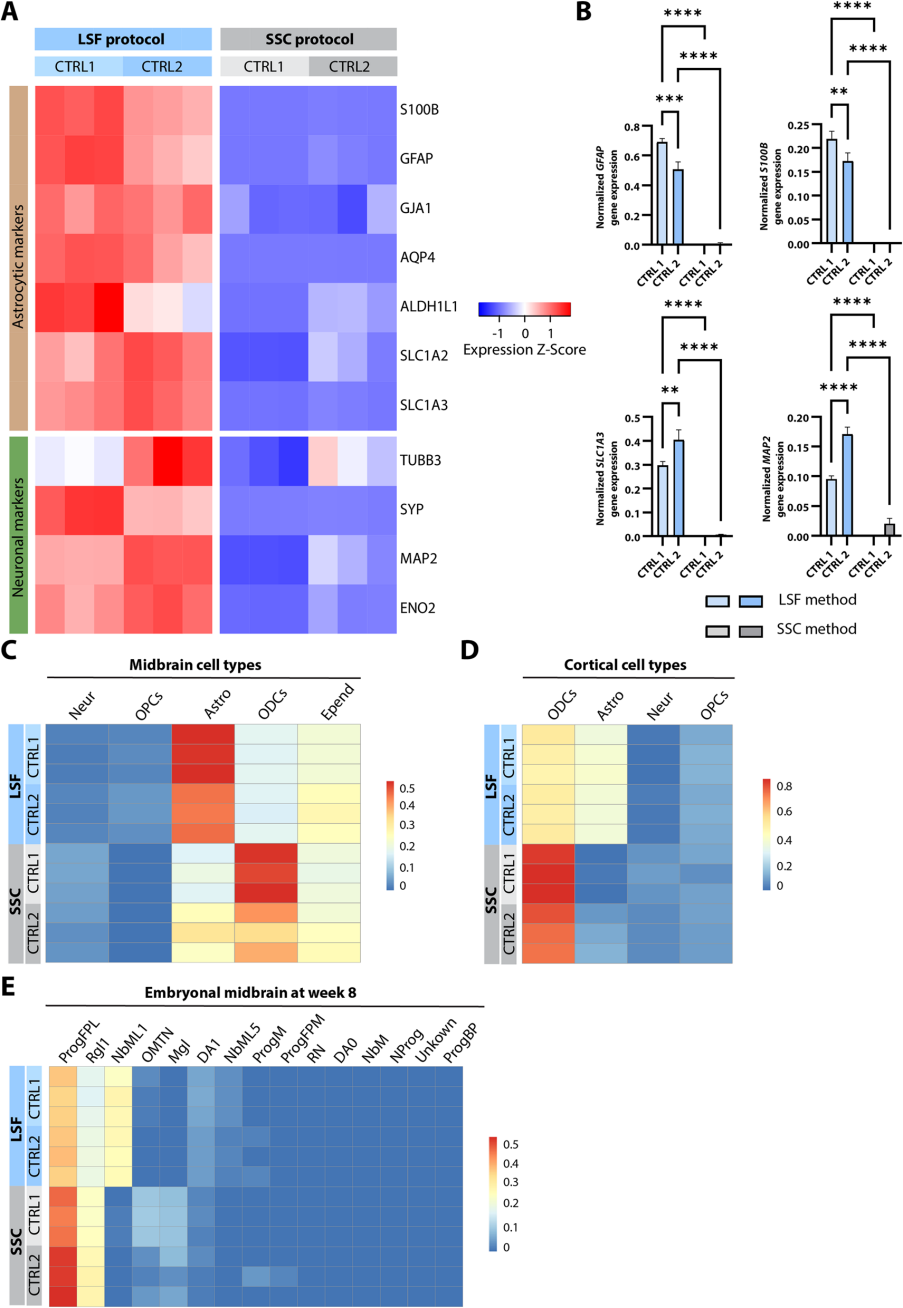
Characterization of cell type composition. **A**. Heatmap displaying expression values for astrocytic and neuronal markers. **B**. Validation of *GFAP*, *S100B*, *SLC1A3,* and *MAP2* expression profiles using qPCR. CTRL1, control 1; CTRL2, control 2. For each protocol and cell line, three biological replicates were used. Data are presented as the mean ± SD,*P ≤ 0.05, **P ≤ 0.01, ***P ≤ 0.001, ***P ≤ 0.0001. **C**. Heatmap showing the percentage of iPSC-derived cells in astrocytic cultures sharing the expression profile with the cell types identified in Smajic et al. [41]. **D**. Heatmap showing the percentage of generated astrocytic cultures sharing the expression signatures with the cortical cell types identified in Agarwal et al. [40]. **E**. Heatmap displaying the comparison of the generated astrocytes with the dataset from human embryonal midbrain at week 8 [42]. CTRL1, control 1; CTRL2, control 2; Mgl, microglia; Astro, astrocytes; Epend, ependymal cells; ODCs, oligodendrocytes; Neur, neurons; OPCs, oligodendrocyte precursor cells; ProgFPL, lateral floorplate progenitor; Rgl1, radial glia-like cell type 1; NbML1, mediolateral neuroblast type 1; OMTN, oculomotor and trochlear nucleus; DA1, dopaminergic neurons 1; NbML5, mediolateral neuroblast type 5; ProgM, midline neuronal progenitor; ProgFPM, medial floorplate progenitor; RN, red nucleus; DA0, dopaminergic neurons 0; NbM, medial neuroblasts; NProg, neuronal progenitor; ProgBP, basal plate progenitor

Next, we assessed astrocytic maturity and the purity of the cultures by analyzing the expression of commonly used astrocytic and neuronal marker genes [13, 14, 49]. Generally, LSF astrocytes presented a higher expression of astrocytic markers, however, the same tendency was shown for neuronal markers (Fig. 4A). We confirmed our findings by quantifying multiple analyzed targets by means of qPCR (Fig. 4B). Interestingly, the expression of mature astrocytic markers such as *AQP4*, *SLC1A3* and *ALDH1L1*, was comparatively upregulated in LSF astrocytes, suggesting that cells attain a more advanced maturation status under these culture conditions.

To further characterize the cultures, we employed the bulk tissue cell type deconvolution method “MuSiC” [39]. Using this approach and applying multiple published human datasets [40–42], we were able to estimate the percentage of cells sharing the characteristics of various brain cell types. When compared to data obtained from postmortem midbrain, LSF cultures showed a higher proportion of cells resembling human mature astrocytes (Fig. 4C). Similarly, LSF cells more closely resembled cortical astrocytes from the human brain than the cells obtained with the SSC protocol (Fig. 4D). Of note, when assessing the cell composition of cultures obtained with the LSF protocol, we did not identify cells that matched the gene expression profile of mature neurons as identified in postmortem midbrain scRNA-seq studies [40, 41]. Regarding the cells generated with SSC protocol, they resembled to a high extent the expression profile of oligodendrocytes, both cortical and midbrain ones.

Thus, we hypothesized that the higher expression of neuronal markers in LSF cultures might be caused by the presence of neuronal precursors rather than highly developed neurons. For a more detailed assessment of the cell type composition, we then additionally utilized a dataset generated from human embryonal midbrain tissue [42]. Based on the expression profiles of embryonal midbrain at week 8 of development, we identified a higher proportion of cells resembling neuroblasts with the LSF compared to the SSC protocol (Fig. 4E). Furthermore, the LSF protocol yielded a higher proportion of cells resembling radial glia than the cultures obtained with the SSC method (Figure S3A, B).

### LSF and SSC astrocytes differ in their activation profiles

**Fig. 5:**
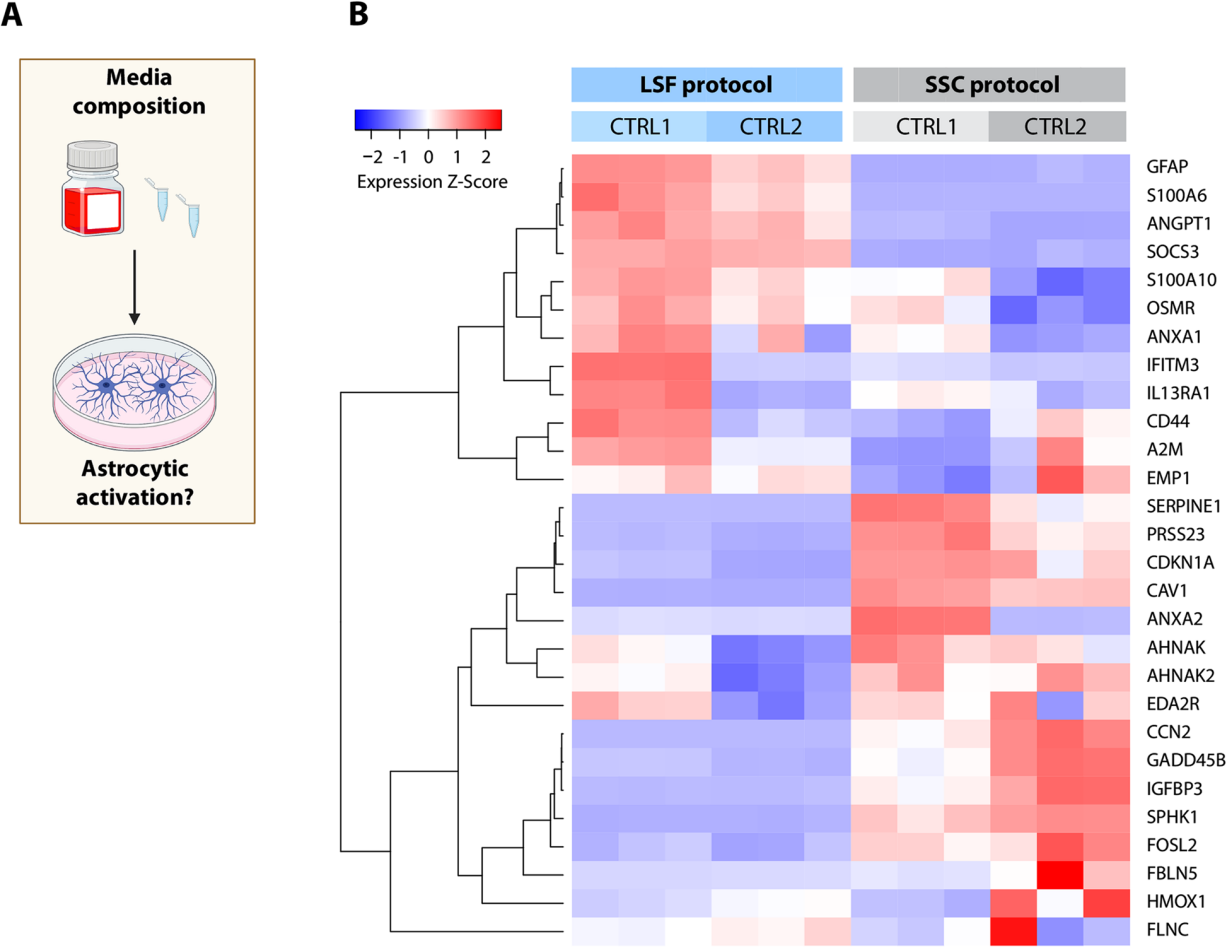
Activation status of astrocytes generated in the study using cell culture media with and without FBS. **A**. Scheme visualizing the research idea. **B**. Heatmap representing the expression Z-score values for genes that were among a core enrichment set identified in astrocytic cultures grown in the presence of FBS as shown in a paper by Magistri and colleagues [50]. Part of the figure was prepared using Biorender

The two applied protocols differ greatly in their media composition used to generate astrocytes. Since it has been reported that FBS presence can lead to astrocytic activation [50, 51] (Fig. 5A), we analyzed the expression of several genes, which have been associated with this phenomenon in previous studies [50, 51]. Using gene set enrichment analysis, Magistri and colleagues identified a set of marker genes that were upregulated in iPSC-derived astrocytes in response to FBS exposure [50]. In line with serum-induced astrocytic activation, 16 of these genes were upregulated in SSC compared to LSF cultures. By contrast, for 12 marker genes, we observed an increase in expression in LSC astrocytes. Of note, this included genes such as *GFAP* and *CD44*, which are known to change their expression not only during astrocytic reactivity but also during cell maturation [44] (Fig. 5B).

## Discussion

With the advent of iPSC technology and the growing recognition of the importance of astrocytes in the pathogenesis of PD, the number of available protocols used to generate iPSC-derived astrocytes is growing [19, 20]. In our study, we aimed to compare two of such methods to understand what their potential advantages and disadvantages could be. The first method, referred to as LSF protocol, is one of the most widely employed astrocyte differentiation methods based on long-term expansion of astrocytic progenitors generated from iPSCs [24, 25] (both papers together were cited more than 500 times to date). The second method, i.e. the SSC protocol, is a straightforward differentiation method that was established in-house and which uses NPCs as the starting point together with FBS-containing medium for astrocytic differentiation and maturation [26]. This protocol has been developed so that the same NPCs, which can be expanded robustly, can be used to generate midbrain dopaminergic neurons as well as astrocytes, facilitating neuron-astrocyte co-culture studies in PD research. Both protocols vary greatly in their duration and yield of generated cells, posing the question about their applicability for different scientific projects. Therefore, we performed a comprehensive imaging and transcriptomic analysis of both methods to unravel the morphological and gene expression profiles of these astrocytes.

Human mature astrocytes possess a distinctive morphology that allows differentiating between cellular soma and highly ramified processes, which account for 80% of the cell volume [45]. The finest processes, known as perisynaptic astrocytic processes (PAPs), develop later during cell maturation [52] and were shown to play a role in an array of brain functions, most importantly in synapse function and maturation [53–55]. Astrocytes are subject to profound changes during reactive gliosis, a phenomenon in which the processes were reported to increase the thickness of their main branches, accompanying an enlargement of the cell soma [56, 57]. High-content imaging analyses of iPSC-derived astrocytes generated in this study, revealed remarkable morphological changes between both protocols. LSF astrocytes presented a more pronounced stellate shape with smaller soma but an increased relative area of the processes. The increased somal area and less defined shape observed for Palm astrocytes (particularly for CTRL2) is in line with a fibroblast-like morphology previously reported for human astrocytes grown in FBS-containing medium [50]. Furthermore, for all morphological parameters assessed in the study, there was a higher consistency between the cell lines for the LSF protocol. Image analysis also revealed a higher percentage of GFAP^+^ cells in LSF astrocytes, while the levels of the early astrocytic marker Vimentin were comparable between the cultures. GFAP is a widely recognized astrocytic marker [58] used to assess differentiation efficiency [59]. Thus, the observed shift in the GFAP to Vimentin ratio in the SSC cultures suggests that these cells are less mature [44]. This finding was supported by gene expression analysis of mature astrocyte markers [14], which were consistently elevated in LSF astrocytes. However, in cells generated with the LSF method, we could also identify a comparatively higher expression of several neuronal markers, suggestive of a contaminant neuronal population.

To study the cellular composition of the generated cultures in more detail, we applied bulk tissue cell type deconvolution using the MuSiC method [39]. With this approach, we compared our bulk RNA-seq data with several datasets produced using scRNA- or snRNA-seq [40–42]. In general, we could observe a higher similarity between cells generated with the LSF protocol and postmortem human astrocytes. The similarity was particularly striking when compared with midbrain astrocytes. These results further strengthen the notion that LSF astrocytes are more mature than SSC cells. Our findings suggest that the LSF protocol is of particular interest for disease modeling, in which mature and functional models are preferred to avoid masking disease phenotypes [60]. This protocol also showed increased reproducibility between the distinct lines used, as demonstrated by similar morphology and percentage of obtained GFAP^+^ cells. However, a disadvantage of this method was the identification of a residual non-glial population based on the expression of neuronal marker genes. Despite this fact, LSF cultures did not show a high degree of similarity to any mature neuronal populations identified in the postmortem datasets. This discrepancy could be due to the changed activation status of LSF cells, as reactive astrocytes were shown to express MAP2 [61]. We also hypothesized that the expression of neuronal markers in these cultures could rather be explained by the presence of immature neuronal progenitors. Indeed, we observed a portion of cells in the LSF cultures that resembled radial glia and neuroblasts as identified in the embryonal datasets [42]. This cellular heterogeneity within the iPSC-derived cultures poses a major challenge for scientists aiming at a high reproducibility of their research. Nevertheless, our microscopy data indicate that the LSF protocol yields over 74% of GFAP^+^ cells.

Next, we assessed the activation status of the generated astrocytes. FBS usage in the astrocytic differentiation protocols was frequently criticized as a driving factor for astrocytic reactivity [50]. Furthermore, FBS is prone to batch inconsistencies and its unknown amount of growth factors and hormones might lead to lower experimental reproducibility [23]. Therefore, we utilized a previously published set of genes [50, 51], which was identified as the core enrichment among tested markers associated with astrocytic activation in response to FBS exposure [50]. In line with serum-induced astrocytic activation, SSC astrocytes showed elevated expression levels for the majority of analyzed marker genes. However, to our surprise, for a subset of marker genes, the mRNA abundance was higher in LSF astrocytes. While this would suggest that even under serum-free conditions, iPSC-derived astrocytes may be activated at baseline, it is worth nothing that this subset included marker genes such as *GFAP* and *CD44,* which are also considered maturation markers and may thus simply indicate a more mature state of LSF astrocytes as already suggested by the aforementioned imaging and MuSiC data [62].

Disease modeling is an important application of iPSC-derived cultures. Together with the technological development of high-throughput devices for drug screening, iPSC-derived cellular models have the potential to advance personalized medicine approaches for neurodegenerative disorders [63]. However, to harness the full potential of iPSC-derived cells in drug screenings, the fast generation of highly homogenous cultures would be a major advantage. The two protocols analyzed here differ greatly in their efficiency and workload required to generate human astrocytes. While the SSC method produces a considerable number of cells in a relatively short time (2 months), the LSF protocol requires prolonged culturing of cells (for 5–6 months) together with manual cutting of the produced spheres. This experimental challenge will likely hamper a potential automation of LSF cultures, whereas SSC astrocytes might be easily obtained using automated platforms [64]. Nevertheless, the LSF protocol seems to be more suitable for initial disease modeling, given the high degree of maturity of the resulting cells as shown in this study.

However, our study also has limitations that should be considered. While we could observe profound differences between the two protocols with respect to astrocyte maturation, we only investigated two control lines in parallel. Thus, our results may have been impacted by this small sample number. Moreover, given that both protocols have previously been used to generate functional astrocytes that were shown to uptake glutamate [26, 65], we did not re-assess their metabolic profiles as part of this study. Instead, we focused on the activation status and maturation of the cells. Future investigations exploring metabolic differences between the protocols (possibly even in a neuron-astrocyte or microglia-astrocyte co-culture setup) are warranted. Finally, it is important to note that the available astrocyte differentiation methods are constantly evolving. Due to this situation, the advantages and disadvantages of various protocols will need to be reassessed in a continuous manner.

Taken together, we showed that astrocytes generated with the LSF method express typical astrocytic markers and resemble their postmortem human counterparts to a higher extent than cells obtained with the SSC protocol. However, the cultures are not purely astrocytic and their generation is more time-consuming, which makes them less suitable for drug screens. We presented an extensive transcriptomic comparison of the protocols, which will provide researchers with relevant information, when choosing the optimal protocol for their research questions.

### Supplementary Information

**Additional file 1.**

## Data Availability

The raw data for this manuscript is not publicly available due to its sensitive nature. The datasets analyzed during the current study, including the scripts used during analyses, are publicly available. Detailed information on how to access the data and the code can be found at https://doi.org/10.17881/4jvc-fq34.
