# Supplementary material for: Comparison of two protocols for the generation of iPSC-derived human astrocytes
Source: Biol Proced Online. 2023 Sep 20;25:26. doi: 10.1186/s12575-023-00218-x (PMC10512486; doi:10.1186/s12575-023-00218-x)
Supplement: Supplementary file 1 — Additional file 1. [file 12575_2023_218_MOESM1_ESM.docx]

**Supplementary information**

**Comparison of two protocols for the generation of iPSC-derived human astrocytes**

# Patrycja Mulica^1^, Carmen Venegas^1^, Zied Landoulsi^1^, Katja Badanjak^1^, Sylvie Delcambre^1^, Maria Tziortziou, Soraya Hezzaz, Jenny Ghelfi, Semra Smajic^1^, Jens Schwamborn^1^, Rejko Krüger^1,2^, Paul Antony^1^, Patrick May^1^, Enrico Glaab^1^, Anne Grünewald^1,3,#,^*, Sandro L. Pereira^1,2,#^


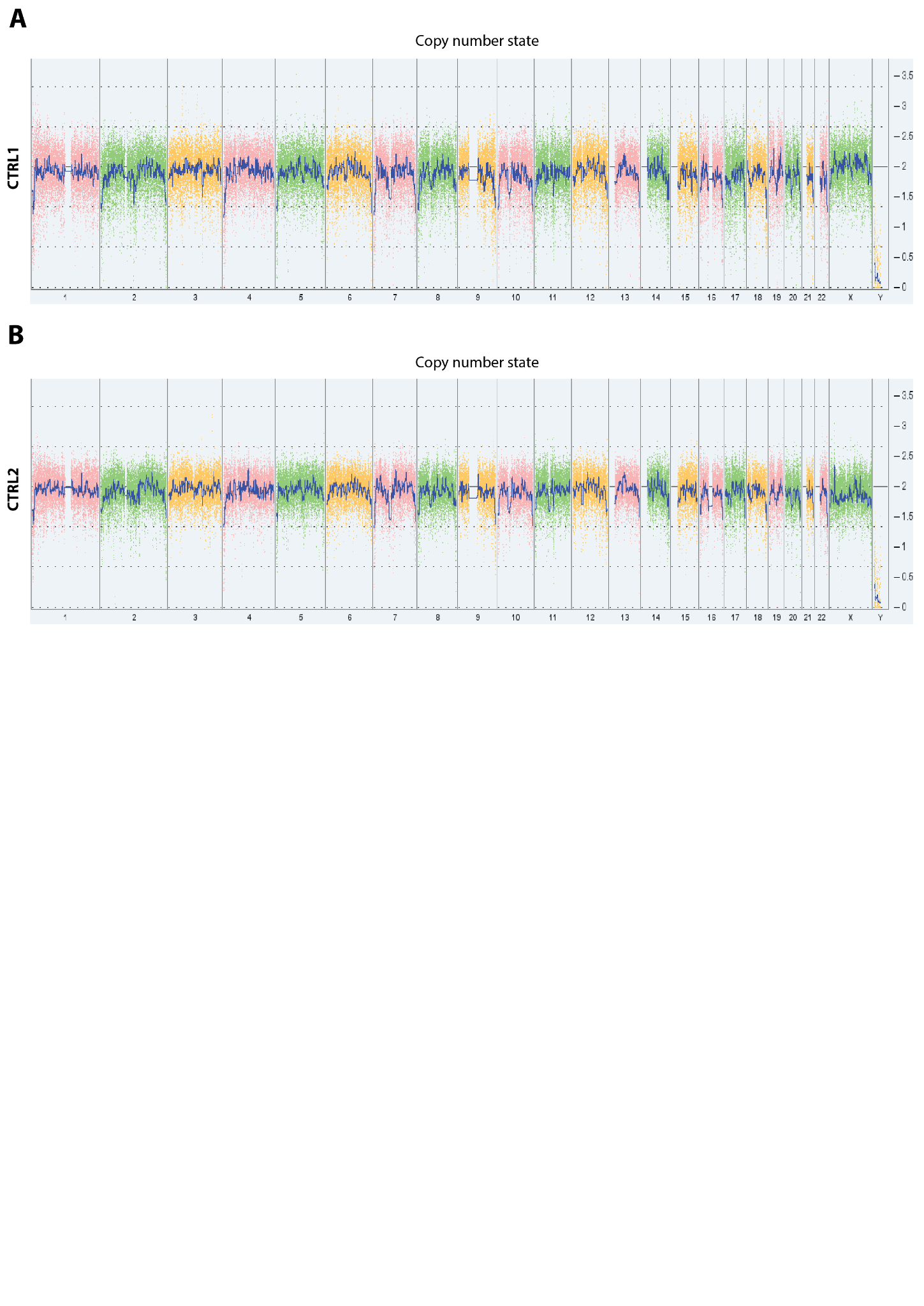


**Figure S1. The analysis of karyotypes.** A. The whole genome view of healthy control 1. B. The whole genome view of healthy control 2. The karyotype analysis was performed using Karyostat^TM^ service from Thermofisher.


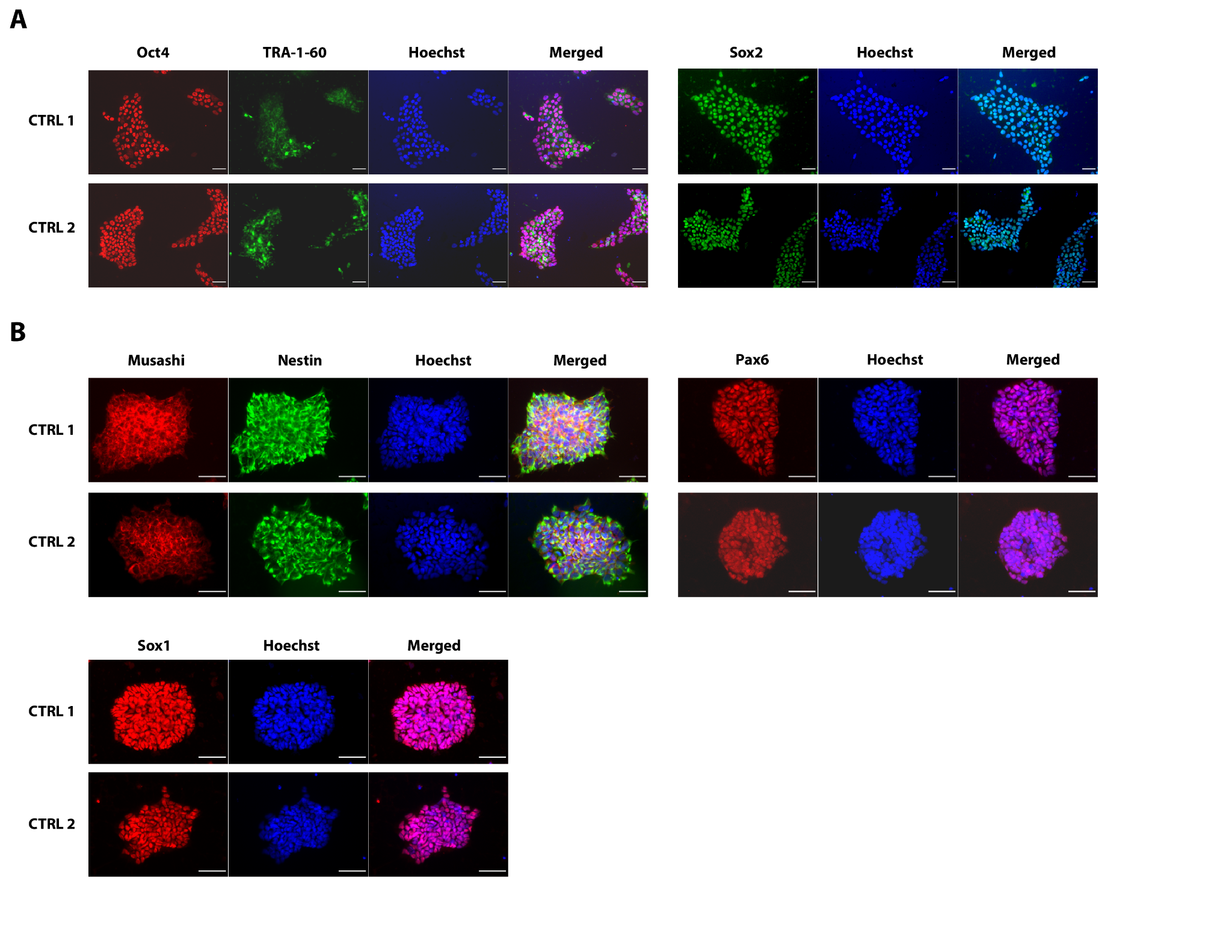


**Figure S2. The analysis of iPSC and NPC markers in immunocytochemistry.** A. Two healthy iPSC controls were analyzed for the expression of Oct4, TRA-1-60 and Sox2. B. Two healthy NPC controls were assessed for the expression of Musashi, Nestin, Pax6 and Sox1. Scale bar: 50 µm.


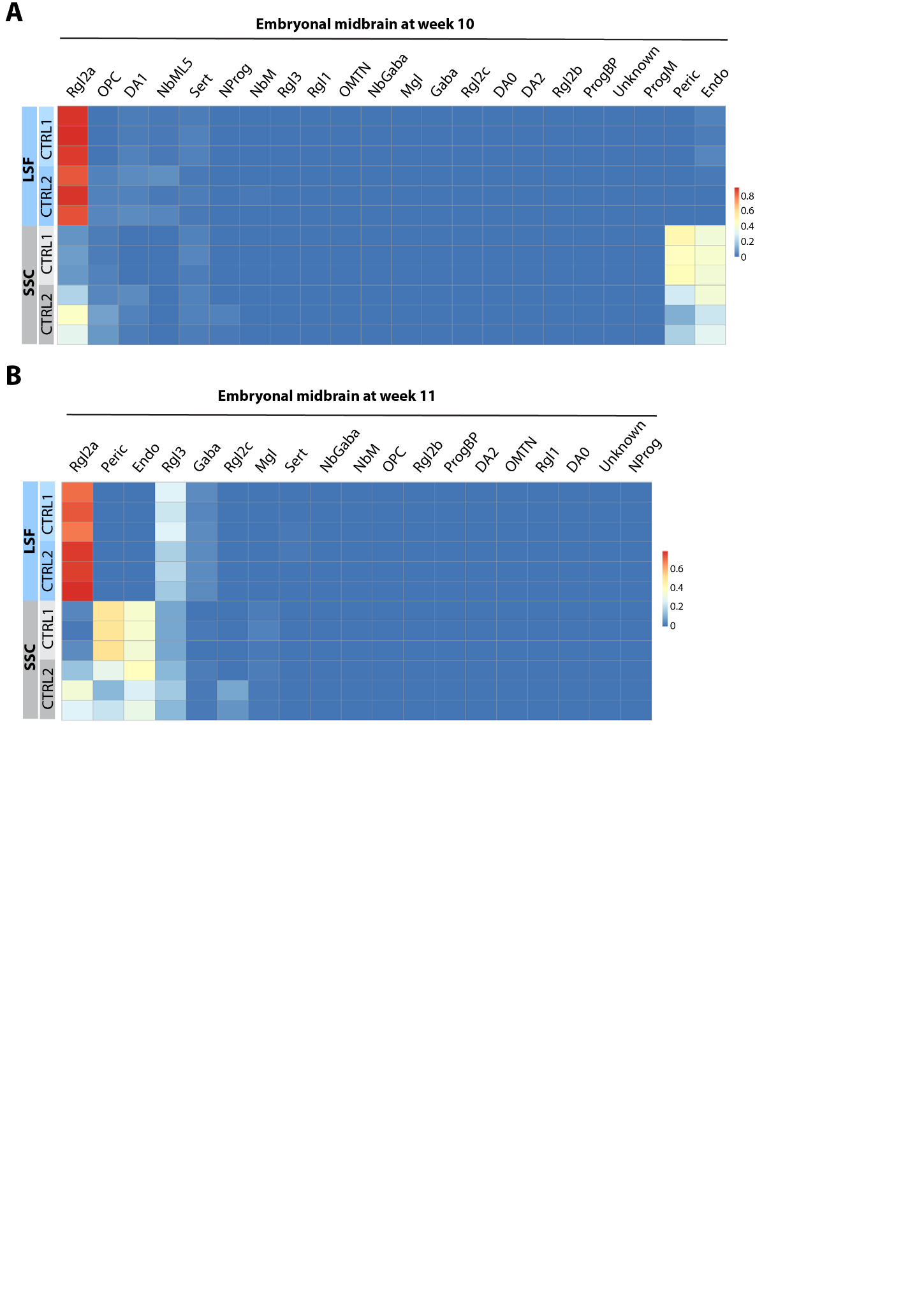


**Figure S3. Heatmaps showing the percentage of cells in the generated cultures resembling cell types identified in human postmortem midbrain and human embryonal midbrains**. As a reference the human embryonal midbrain datasets [[42]](https://www.zotero.org/google-docs/?2JAPko) from week 10 (panel A) and week 11 (panel B) of development. Endo, Endocytes; Gaba, GABAergic neurons; OPC, Oligodendrocyte Precursor Cells; OMTN, oculomotor and trochlear nucleus; NbM, medial neuroblasts; NProg, Neuronal Progenitors; ProgBP, basal plate progenitor; ProgFPL, lateral floorplate progenitor; ProgM, midline neuronal progenitor; Peric, Pericytes; NbML5, mediolateral neuroblasts type 5; DA0, dopaminergic neurons 0; Rgl1, radial glia type 1; DA1, dopaminergic neurons 1; Rgl2a, radial glia type 2a; Rgl2b, radial glia type 2b; Mgl, microglia, NbGaba, neuroblasts GABAergic; Sert, serotonergic neurons; DA2, dopaminergic neurons 2; Rgl3, radial glia type 3; Rgl2c, radial glia type 2c.
